# Adulthood blood levels of hsa-miR-29b-3p associate with preterm birth and adult metabolic and cognitive health

**DOI:** 10.1038/s41598-021-88465-4

**Published:** 2021-04-28

**Authors:** Saara Marttila, Suvi Rovio, Pashupati P. Mishra, Ilkka Seppälä, Leo-Pekka Lyytikäinen, Markus Juonala, Melanie Waldenberger, Niku Oksala, Mika Ala-Korpela, Emily Harville, Nina Hutri-Kähönen, Mika Kähönen, Olli Raitakari, Terho Lehtimäki, Emma Raitoharju

**Affiliations:** 1 Department of Clinical Chemistry, Pirkanmaa Hospital District, Fimlab Laboratories, and Finnish Cardiovascular Research Center, Tampere, Faculty of Medicine and Health Technology, Tampere University, Tampere, Finland; 2 Gerontology Research Center, Tampere University, Tampere, Finland; 3 Centre for Population Health Research, University of Turku and Turku University Hospital, Turku, Finland; 4 Research Centre of Applied and Preventive Cardiovascular Medicine, University of Turku, Turku, Finland; 5 Division of Medicine, Turku University Hospital and Department of Medicine, University of Turku, Turku, Finland; 6 Research Unit Molecular Epidemiology, Helmholtz Zentrum Munich, German Research Center for Environmental Health, Munich, Germany; 7 Vascular Centre, Tampere University Hospital, Tampere, Finland; 8 Computational Medicine, Faculty of Medicine, University of Oulu, Oulu, Finland; 9 Center for Life Course Health Research, Faculty of Medicine, University of Oulu, Oulu, Finland; 10 Biocenter Oulu, University of Oulu, Oulu, Finland; 11 NMR Metabolomics Laboratory, School of Pharmacy, University of Eastern Finland, Kuopio, Finland; 12 Department of Epidemiology, School of Public Health and Tropical Medicine, Tulane University, New Orleans, LA USA; 13 Department of Pediatrics, Tampere University and Tampere University Hospital, Tampere, Finland; 14 Department of Clinical Physiology, Tampere University Hospital, and Faculty of Medicine and Health Technology, Tampere University, Tampere, Finland; 15 Department of Clinical Physiology and Nuclear Medicine, University of Turku and Turku University Hospital, Turku, Finland

**Keywords:** Gene expression, Gene regulation, miRNAs, Type 2 diabetes

## Abstract

Preterm birth (PTB) is associated with increased risk of type 2 diabetes and neurocognitive impairment later in life. We analyzed for the first time the associations of PTB with blood miRNA levels in adulthood. We also investigated the relationship of PTB associated miRNAs and adulthood phenotypes previously linked with premature birth. Blood MicroRNA profiling, genome-wide gene expression analysis, computer-based cognitive testing battery (CANTAB) and serum NMR metabolomics were performed for Young Finns Study subjects (aged 34–49 years, full-term n = 682, preterm n = 84). Preterm birth (vs. full-term) was associated with adulthood levels of hsa-miR-29b-3p in a fully adjusted regression model (p = 1.90 × 10^–4^, FDR = 0.046). The levels of hsa-miR-29b-3p were down-regulated in subjects with PTB with appropriate birthweight for gestational age (p = 0.002, fold change [FC] = − 1.20) and specifically in PTB subjects with small birthweight for gestational age (p = 0.095, FC = − 1.39) in comparison to individuals born full term. Hsa-miR-29b-3p levels correlated with the expressions of its target-mRNAs BCL11A and CS and the gene set analysis results indicated a target-mRNA driven association between hsa-miR-29b-3p levels and *Alzheimer's disease*, *Parkinson's disease*, *Insulin signaling* and *Regulation of Actin Cytoskeleton* pathway expression. The level of hsa-miR-29b-3p was directly associated with visual processing and sustained attention in CANTAB test and inversely associated with serum levels of VLDL subclass component and triglyceride levels. In conlcusion, adult blood levels of hsa-miR-29b-3p were lower in subjects born preterm. Hsa-miR-29b-3p associated with cognitive function and may be linked with adulthood morbidities in subjects born preterm, possibly through regulation of gene sets related to neurodegenerative diseases and insulin signaling as well as VLDL and triglyceride metabolism.

## Introduction

Preterm birth (PTB), defined as birth of an infant at less than 37 weeks’ gestational age associates with increased risk of developmental defects and mortality in infants as well as multiple diseases later in life^1^. In addition to PTB, growth restriction and being born small for gestational age have been shown to increase the risk of multiple maladies^2^. Altogether, PTB subjects are at greater risk of neurodevelopmental disorders, have worse academic performance at school and poorer performance in childhood cognitive tests^3–5^. Cohort studies from Nordic countries have also indicated that late PTB (34 weeks 0 days to 36 weeks 6 days of gestation) is associated with poorer episodic memory performance in adulthood, increased risk of neurodegenerative diseases^6^ and risk of accelerated age related cognitive dysfunction^7^. Subjects born preterm have been shown to have increased prevalence of chronic diseases, such as asthma^8^, type 1 and type 2 diabetes (T2D)^9^, and cardiovascular risk factors and decreased arterial health^10–12^. The effect of PTB on later life health is conveyed by malformations associated with PTB^13^; altered pattern of growth, increased whole body adiposity, and partitioning of adipose tissue^14,15^; and subtle abnormalities of central nervous system function^16^. It has also been suggested that intrauterine conditions and PTB affect individuals’ health through changes in epigenetic factors, namely, histone modifications^17^, DNA-methylation profiles^18,19^, and non-coding RNAs^20^. Prenatal insults and stress can affect individuals’ epigenome and long-term gene expression regulation, which in turn can convey the increased risk of chronic diseases later in life^21^.

MicroRNAs (miRNAs, miRs) are small non-coding RNAs that primarily regulate gene expression by binding to target mRNAs and interfering with the translation^22,23^. In addition to translational repression, miRNAs have been shown to function as translational^24^ and transcriptional activators, the latter of which may happen via epigenetic modulation^25^. MicroRNAs can also be transported between cells and tissues via circulation. These circulating miRNAs have been shown to participate in cell-to-cell communication^26^, potentially contributing to disease progression, but also have been shown to act as indicators for pathological processes elsewhere in the body.

Changes in miRNA profiles have been associated with many chronic diseases with partly developmental origins, such as asthma^27^, T2D^28^ and cardiovascular diseases^29^. Additionally, miRNA expression has been shown to be altered in complications of pregnancy, such as miscarriages^30^ and preeclampsia^31,32^. In animal studies, the nutritional and stress status of the mother has been shown to alter the offspring’s miRNA profile in various tissues and affect the gestational length and insulin sensitivity of the offspring^20,33^. It has also been shown that the miRNA profiles of the placenta^31^, amniotic fluid^34^, and maternal serum^32^, whole blood and monocytes^35^ differ between preterm and full term births. Although the lasting effect on PTB on DNA methylation pattern^19,36^ and subjects’ health^37^ have been studied, only a few studies analyzing the associations of PTB with the subjects’ circulatory miRNA profile have been conducted on newborns^38–41^ and one case–control study (n = 50) with 4 selected miRNAs in young adults^42^.

Our hypothesis is that PTB is associated with the blood levels of miRNAs in adulthood and that this altered miRNA expression may associate with increased risk of chronic diseases in prematurely born subjects later in their lives. More specifically, we aimed to (1) analyze the differences in blood miRNA levels in adulthood between subjects born full term (FTB) and preterm or alternatively PTB with appropriate birth weight for gestational age (AGA) or PTB with small birth weight for gestational age (SGA), (2) discover blood target mRNAs affected by these dysregulated miRNAs, (3) research the biological gene sets over the whole transcriptome that are affected by these miRNAs (4) and to investigate the possible association of these dysregulated miRNAs and adulthood indices of those conditions previously associated with PTB, such as T2D, dyslipidemias and lower cognitive function.

## Methods

More detailed materials and methods can be found in the Additional File 1.

### The Young Finns Study (YFS)

YFS is a multicenter follow-up study on cardiovascular risk factors from childhood to adulthood in Finland. The YFS was launched in 1980, when 3,596 children and adolescents (3–18 years old) participated in the baseline study^43^. Thereafter, the subjects have been followed up with several examinations including comprehensive risk factor assessments. The 30-year follow-up was conducted in 2011, with 2,063 adults, aged 34–49 years, participating in the study. The examinations included physical measurements, blood tests, and questionnaires. All measurements utilized in this study, excluding birthweight, prematurity at birth and parental education level at baseline, are derived from the 2011 follow-up study and samples collected during it. The miRNA profiling population comprises 871 subjects from the 2011 follow-up study. When the population in the miRNA analysis was compared to the whole 2011 follow-up study population, the only difference was observed in the prevalence of T2D, which in the whole population is 6.0% and in the miRNA profiling cohort only 3.0%. Birth weight and preterm status were available for 761 subjects in the miRNA profiling sub-population. The demographics are presented in Supplementary Table S1.

### Fetal growth and PTB

PTB status was defined as birth before 37 weeks’ gestation, and the number of weeks preterm was ascertained in those who reported PTB. Birthweight was self-reported during the follow-up studies in 1983 and 1986. Subjects born preterm were categorized as either AGA or with SGA using a cut point of − 1 SD z score (corresponding to the 15th percentile) based on Finnish sex and gestational age-stratified birthweight percentiles^12^. AGA describes a newborn infant whose size is within the normal range for his or her gestational age, while newborns with SGA were smaller than expected for their gestational weeks. The study population did not include any subjects born preterm and large for their gestational age.

### RNA isolation, miRNA and gene expression profiling

RNA was isolated and miRNA and gene expression profiling was performed as described before^44^. Briefly, whole blood was collected in PaXgene Blood RNA Tubes (PreAnalytix) and isolated with a PAXgene Blood microRNA Kit (Qiagen) including the DNase Set using the QiaCube according to the manufacturer’s instructions.

As described before in^44^, microRNA expression profiling was performed with the TaqMan MicroRNA Panel (Applied Biosystems) containing 758 microRNAs using the standard protocol. MicroRNA panels were loaded using the AccuFill System and run with the QuantStudio 12K Flex (Applied Biosystems). Primary data analysis was performed with Expression Suite Software version 1.0.1. U6 snRNA, RNU44, and RNU48 were used as housekeeping small RNAs. 243 miRNAs that were expressed in at least 2/3 of the samples were included in further analysis. After quality control and removal of outlier miRNA, profiling was successful on 871 samples. To correct for batch effects, principal components analysis was performed for miRNA expression data. The data was adjusted for 10 of the first 20 principal components from the principal component analysis.

As described in^44^, the expression levels were analyzed with an Illumina HumanHT-12 version 4 Expression BeadChip (Illumina Inc.). The BeadChips were scanned with the Illumina iScan system. The expression data was processed using nonparametric background correction, followed by quantile normalization with control and expression probes, using the neqc function in the limma package and log2 transformation. The expression analysis was successful in 743 of the 871 samples with a miRNA expression profile.

### Clinical and biochemical measurements

As previously described^44^, weight, height, waist circumference, and blood pressure were measured, and body mass index (BMI) was calculated. Blood cell parameters were measured with flow cytometric particle counting and photometry. The serum triglyceride, glucose, and total cholesterol concentrations were analyzed using the enzymatic methods. High density lipoprotein (HDL) cholesterol levels were estimated after the precipitation of apolipoprotein B-containing lipoproteins. Low density lipoprotein cholesterol was calculated indirectly using the Friedewald formula. For glycated hemoglobin (HbA1c) fraction measurement, the concentration of total hemoglobin was determined colorimetrically, after which the concentration of HbA1c was measured immunoturbidimetrically. These two concentrations were used to calculate the HbA1c percentage (HbA1c%). Insulin levels were measured by a microparticle enzyme immunoassay kit. Subjects were categorized into the normoglycemic, impaired fasting glucose, and T2D groups, based on fasting serum glucose and HbA1c according to the criteria of the WHO^45^ and self-reported diagnosis of T2D by a physician. Subjects with type 1 diabetes were excluded from the analysis.

### NMR metabolomics

A high-throughput serum NMR metabolomics platform was used for absolute quantification of serum lipids and metabolites, including lipoprotein subclass distributions, fatty acids, and various small molecules such as amino acids and glycolysis precursors^46,47^. The analyzed 14 lipoprotein subclasses were defined based on particle size. The details of the experimentation have been described^46–48^. Data were available from all the subjects with successful miRNA profiling.

### Cognitive function

A test battery developed by the Cambridge Cognition (CANTAB) was used to assess cognitive function among the subjects in the follow-up study in 2011. The CANTAB test is a computerized, predominantly non-linguistic and culturally neutral test focusing on a wide range of cognitive domains. The test was performed using a validated touch-screen computer system and was successful on all the 871 subjects with miRNA expression data. In YFS, the test battery was compiled and performed as described earlier^49^. During cognitive testing the participants conducted a Motor screening test, Paired associates learning (PAL) test, Spatial working memory (SWM) test, Reaction time (RTI) test and Rapid visual information processing (RVP) test. Each test produced several variables. Principal component analysis was conducted for each test to identify components accounting for the majority of the variation within the dataset. The first principal component was selected to represent the performance in each separate test. After distribution analyses, the Motor screening test component was excluded from further analyses due to ceiling effect. Other components were normalized using rank order normalization procedure resulting in four variables (mean 0, standard deviation (SD) 1)^49^.

### Statistical analysis

Association between PTB status (full term birth vs. preterm birth) and adulthood miRNA levels was analyzed with linear regression using age, sex, smoking (yes/no), serum insulin and glucose levels, leucocyte, erythrocyte and thrombocyte count and liver status (fatty liver yes/no) as covariates (MODEL 1). To account for multiple testing, only miRNAs with FDR (Benjamini-Hochberg) < 0.05 were further investigated. Median fold changes (FC) between different birth status groups (FTB, PTB with AGA or PTB with SGA) were calculated and pairwise statistical significance was evaluated with Mann–Whitney U test and over all groups using Kruskal–Wallis test for trend.

The flow and results of transcriptomic and gene set analysis are presented in the Supplementary Fig. S1. The predicted mRNA targets of miRNAs of interest were included in the correlation analysis if they were recognized by one or more in silico miRNA target prediction programs in miRGator^50^. Correlations between miRNAs of interest and their predicted targets were calculated with Spearman’s correlation and individual correlations with FDR < 0.05 (both positive and negative correlations) were considered significant. Predicted target mRNAs with correlation at the level of FDR < 0.25 with the levels miRNA of interest were selected in further analysis. The overlaps between the selected target mRNAs and gene set in KEGG^51^ and BIOCARTA were analyzed in molecular signature database. Gene sets containing at least 5 of the selected target mRNAs and with FDR < 0.05 for the enrichment were included in gene set enrichment analysis (GSEA). In GSEA (adjusted for age and sex), the association between the expression of all genes in the identified gene sets and blood levels of the miRNA of interest were evaluated and FDR < 0.25 was considered significant.

Association between the miRNAs of interest and the metabolite levels as well as physiological features previously associated with metabolic dysfunction (listed in^44^) were analyzed one by one with linear regression analysis using age, sex, smoking, birth status, leucocyte, erythrocyte and thrombocyte count, fatty liver status and polygenic risk score for metabolic syndrome^52^ as covariates (MODEL 2). Associations between the miRNA and diagnosis of hypertension, T2D and impaired fasting glucose were analyzed separately with binominal regression models using the covariates according to the MODEL 2.

The associations between the miRNA of interest and each studied cognitive domain were analyzed separately with linear regression with age, sex, smoking, birth status, leucocyte, erythrocyte and thrombocyte count and fatty liver status as well as parental education years (less than 9 years, 9–12 years or more than 12 years), subject’s own education level (participated in higher academic education yes/no) and polygenic risk score for cognitive function as covariates (MODEL 3). If the miRNA of interest associated significantly (FDR < 0.05) with any of the cognitive domains, its association with the individual variables produced by the specific cognitive test for that particular cognitive domain was also analyzed one by one utilizing the adjustments according to the MODEL 3. FDR < 0.05 was considered significant when utilizing models 1, 2 and 3.

### Ethics approval and consent to participate

The present study has been approved by the 1st ethical committee of the Hospital District of Southwest Finland on September 21st, 2010 and by local ethical committees. All study subjects gave an informed consent, and the study was conducted according to the principles of the Declaration of Helsinki.

## Results

### Insulin levels and prevalence of impaired fasting glucose are higher in subjects with PTB

Our data comprises of 681 subjects with FTB, 67 with PTB and AGA and 17 with PTB and SGA (Supplementary Table S1). The prevalence of impaired fasting glucose was higher in subjects with PTB vs FTB (chi square test, p = 0.039). Difference was detected also when comparing only the subjects with PTB and SGA to those with FTB (p = 0.046). In line with these results, insulin levels were also higher in the subjects with PTB in comparison to those with reported FTB (p = 0.042). In addition to these indicators of impaired glucose metabolism, we observed higher thrombocyte count on the subjects with PTB and AGA in comparison to those with FTB (p = 0.043). The median BMI, and the prevalence of fatty liver and female sex were higher in the subjects with PTB and SGA than in the FTB subjects, but most likely due to the small number of subjects with PTB and SGA, these differences were statistically non-significant.

### Adulthood hsa-miR-29b-3p levels are lower in PTB subjects than in subjects born full term

**Figure 1:**
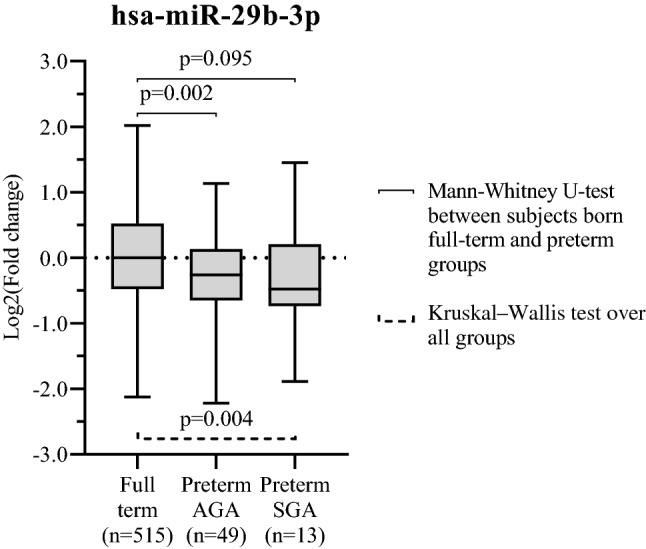
Blood levels of hsa-miR-29b-3p in subjects born preterm in comparison to those born full term. Blood levels of hsa-miR-29b-3p of subjects born preterm and either appropriate birth weight for gestational age (AGA) or small birth weight for gestational age (SGA) described by the Log2(fold changes) in comparison to subjects born full-term. MicroRNA-29b-3p expression was detected in 551 participants with birth weight and birth status information.

Of the analyzed 243 miRNAs, adult blood levels of hsa-miR-29b-3p, -409-3p and -21-5p were nominally associated with birth status (FTB vs. PTB) in MODEL 1 (Supplementary Table S2). Only blood levels of hsa-miR-29b-3p were associated with PTB after multiple testing correction (p = 1.90 × 10^–4^, FDR = 0.046, β = − 0.521 95% CI − 0.792 to − 0.249) in this model. When comparing non-adjusted miRNA FC, there was a trend over birth status groups (p = 0.002, Fig. 1). Subjects born preterm with AGA had lower levels of hsa-miR-29b-3p than subjects born full-term (FC = − 1.20, p = 0.002) and this down-regulation was quantitatively more substantial (FC = − 1.39, p = 0.095), in subjects born preterm with SGA, compared to subjects born full-term (Fig. 1). Trend of expression was similar for males and females separately, but the association was statistically significant only in women (Supplementary Fig. S2). The levels of hsa-miR-29b-3p were not associated with birthweight in subjects with FTB.

### Levels of hsa-miR-29n-3p in subjects with FTB or PTB in infancy

As we did not have miRNA data from birth from our study subjects, we retrieved data from Rager et al.^53^ from Gene Expression Omnibus (GSE48353), to address the question whether miR-29b-3p levels were lower in the blood of PTB infants at birth in comparison to those with FTB. Even though their data set of miRNA profiling performed for 40 cord blood samples collected from the infants immediately following delivery, contained only two subjects who were born before gestational week 37, their median hsa-miR-29b-3p levels were lower than the median of those who were born at or after gestational week 37 (FC = − 1.82, p = 0.023) (Supplementary Fig. S3).

### Hsa-miR-29b-3p blood levels correlate negatively with BAF chromatin remodeling complex subunit BCL11A (BCL11A) and positively with citrate synthase (CS) mRNA levels

MiRGator v. 3.0 gives a total of 6102 predicted targets for hsa-miR-29b-3p. In our whole blood transcriptomics data hsa-miR-29b-3p levels correlated at the level of FDR < 0.25 with 269 of these predicted targets (Supplementary Table S3 in Additional file 2). These mRNAs were included in the subsequent gene set analysis. After appropriate multiple testing correction, only BCL11A mRNA levels correlated inversely with hsa-miR-29b-3p levels in blood (p = 1.31 × 10^–5^, FDR = 0.035, r = − 0.183). In addition, hsa-miR-28b-3p levels correlated positively with CS mRNA levels (p = 1.90 × 10^–5^, FDR = 0.035, r = 0.180).

### Target mRNA driven whole transcriptome wide gene set analysis for hsa-miR-29b-3p levels pinpoints pathways associated with neurodegenerative diseases and insulin signaling

The 269 correlated targets of hsa-miR-29b-3p were enriched in 13 biological gene sets (Table 1). When performing GSEA for these selected 13 pathways and hsa-miR-29b-3p levels, the expression of *Alzheimer’s* and *Parkinson’s disease* pathways were negatively associated with hsa-miR-29b-3p levels and the expression of *Insulin signaling* pathway and *Regulation of actin cytoskeleton* were associated positively with hsa-miR-29b-3p levels (Table 1).

**Table 1:** Target-mRNA driven gene set enrichment analysis for hsa-miR-29b-3p levels.

Gene set	Target genes enriched in gene sets			Gene sets association with hsa-miR-29b-3p levels		
	p-value	FDR	Genes in overlap	FWER	FDR	Direction of association
Alzheimer's disease	3.83 × 10^–4^	0.014	ADAM10, COX7A2L, COX7C, ITPR1, NDUFA10, UQCRQ	0.237	**0.084**	Negative
Insulin signaling pathway	0.001	0.026	FASN, PPP1CB, PRKX, PYGB, RPS6KB2	0.080	**0.145**	Positive
Parkinson's disease	1.05 × 10^–4^	0.008	COX7A2L, COX7C, NDUFA10, SEPT5, UBE2L3, UQCRQ	0.234	**0.165**	Negative
Regulation of actin cytoskeleton	0.001	0.033	ITGA5, ITGB2, ITGB5, PPP1CB, RAC2, VCL	0.202	**0.234**	Positive
Gap junction	1.51 × 10^–4^	0.008	GNAS, ITPR1, PRKX, TUBA1B, TUBB1	0.511	0.353	Positive
MAPKinase Signaling Pathway^a^	1.29 × 10^–4^	0.008	MAP2K3, MAP2K4, RPS6KA1, RPS6KA3, RPS6KB2	0.560	0.361	Positive
Hematopoietic cell lineage	1.36 × 10^–4^	0.008	CD4, CD44, IL11RA, ITGA5, MS4A1	0.629	0.366	Positive
Cell adhesion molecules (CAMs)	0.001	0.025	ALCAM, CD4, CD58, ITGB2, SELP	0.599	0.369	Positive
GnRH signaling pathway	2.60 × 10^–4^	0.012	GNAS, ITPR1, MAP2K3, MAP2K4, PRKX	0.492	0.416	Positive
Vascular smooth muscle contraction	4.72 × 10^–4^	0.015	GNAS, ITPR1, KCNMB2, PPP1CB, PRKX	0.411	0.429	Positive
MAPK signaling pathway	1.35 × 10^–4^	0.008	MAP2K3, MAP2K4, PPM1B, PRKX, PTPN7, RAC2, RPS6KA1, RPS6KA3	0.760	0.545	Positive
Long-term potentiation	4.56 × 10^–5^	0.008	ITPR1, PPP1CB, PRKX, RPS6KA1, RPS6KA3	0.823	0.639	Positive
Oocyte meiosis	4.54 × 10^–4^	0.015	ITPR1, PPP1CB, PRKX, RPS6KA1, RPS6KA3	0.856	0.674	Negative

Thirteen gene sets were enriched with predicted and correlating (FDR < 0.25) targets of hsa-miR-29b-3p. When performing GSEA for these 13 gene sets and hsa-miR-29b-3p levels, four were associated (FDR < 0.25, in bold) with the expression of hsa-miR-29b-3p. This indicates a possible hsa-miR-29b-3p target mRNA driven regulation of these gene sets. The flow and results of transcriptomics and gene set analysis are presented in Supplementary Fig. S1

*Notes* All gene sets except MAPKinase Signaling Pathway (BIOCARTA^a^) are from KEGG collection. Both gene-expression data and hsa-miR-29b-3p levels were available from 559 study subjects.

*GSEA* gene set enrichment analysis; *FDR* False discovery rate.

### Hsa-miR-29b-3p levels associated with visual processing and sustained attention

From the 4 cognitive domains analyzed, hsa-miR-29b-3p levels were associated directly and independently with visual processing and sustained attention (Rapid visual information processing test; p = 0.005, FDR = 0.020; β = 0.115, 95%CI = 0.035–0.195) (Table 2, MODEL 2). When analyzing the individual variables within the test measuring this cognitive domain, the association was strongest with the total of correct rejections (p = 0.008, FDR = 0.025; β = 0.106, 95%CI = 0.028–0.184).

**Table 2:** Association between hsa-miR-29b-3p levels and cognitive domains and variables indicating visual processing and sustained attention.

	p-value	FDR	β	95% CI
**Cognitive domains**				
Visual processing and sustained attention (RVP test)	0.005	0.020	0.115	0.035–0.195
Episodic memory and associative learning (PAL test)	0.280	0.547	0.043	− 0.035 to 0.121
Short term working memory (SWM test)	0.410	0.547	0.034	− 0.047 to 0.116
Reaction and movement time (RTI test)	0.569	0.569	0.025	− 0.112 to 0.061
**Variables indicating visual processing and sustained attention**				
Total correct rejections	0.005	0.025	0.112	0.034–0.191
Signal detection measure of sensitivity to the target (A’)	0.008	0.025	0.105	0.028–0.184
Total misses	0.013	0.025	− 0.099	− 0.177 to − 0.021
Probability of hit	0.013	0.025	0.098	0.021–0176
Total hits	0.014	0.025	0.098	0.020–0.175
Probability of false alarm	0.041	0.061	− 0.078	− 0.152 to 0.003
Total false alarms	0.070	0.089	− 0.070	− 0.143 to 0.005
Signal detection measure of the strength of trace required to elicit a response (B’)	0.091	0.102	0.068	− 0.011 to 0.146
Mean latency	0.362	0.362	− 0.044	− 0.126 to 0.046

The association between hsa-miR-29b-3p levels and the cognitive domains was analyzed one by one with linear regression with age, sex, smoking, birth status, leucocyte, erythrocyte and thrombocyte count and liver status as well as parental education years, participants own education level and and polygenic risk score for cognitive function as covariates (MODEL 3). All variables were available from 513 subjects. As the principal component of Rapid visual information processing test was associated with hsa-miR-29b-3p levels the association to the individual variables produced by the CANTAB test battery were also analyzed one by one utilizing the same MODEL 3.

*Note* The CANTAB test battery used to determine the cognitive domains including four separate tests: (1) Paired associates learning (PAL) test, (2) Spatial working memory (PWM) test, (3) Reaction time (RTI) test and (4) Rapid visual information processing (RVP) test.FDR, False discovery rate.

### Hsa-miR-29b-3p levels associated with levels of serum very low-density lipoprotein (VLDL) subclasses lipid and triglyceride levels

**Figure 2:**
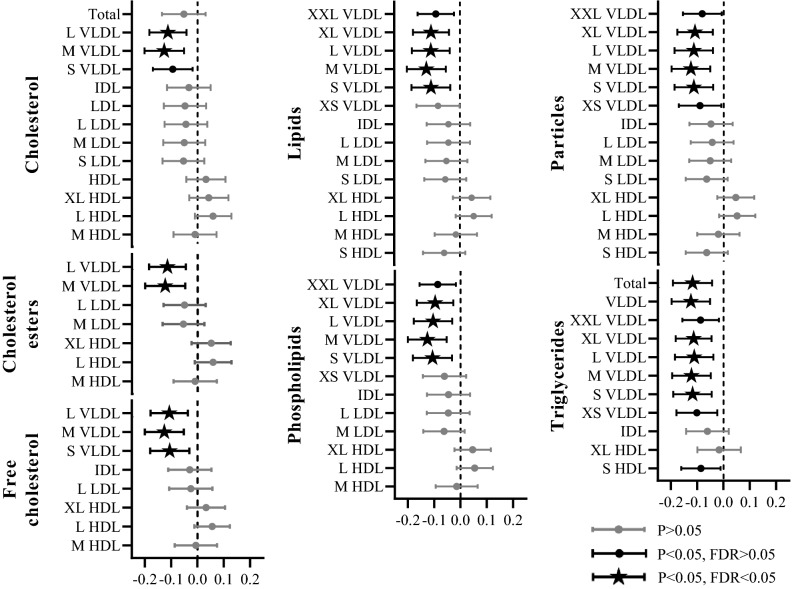
Associations of blood hsa-miR-29b-3p levels with serum lipoprotein subclass particle size and lipid components. The serum lipoprotein subclass particle size and lipid components measured by NMR-spectroscopy. Values are standard deviation (SD) increment in metabolite measure and 95% confidence intervals (95% CI) per one SD change of miRNA of interest are presented. Associations have been analyzed with a linear regression model adjusted with age, sex, smoking, birth status, liver status, polygenic risk score for metabolic syndrome and blood cell counts (MODEL 2). n for the model is 551. *XL* extra large, *L* large, *M* medium, *S* small, *XS* extra small, *HDL* high-density lipoprotein, *LDL* low-density lipoprotein, *IDL* intermediate-density lipoprotein, *VLDL* very-low-density lipoprotein.

Hsa-miR-29b-3p levels inversely and independently associated with the levels of different subclasses of VLDL in MODEL 3, most frequently with those of medium VLDL. From different types of lipoprotein components, the association was most systematically observed with triglyceride levels. In addition to above NMR-metabolic measures, hsa-miR-29b-3p levels were associated with the conventionally measured triglyceride levels (p = 0.003, FDR = 0.023, β = − 0.111, 95%CI = − 0.185 to − 0.037) (Fig. 2, Supplementary Table S4). There was no association between the diagnosis of hypertension, impaired fasting glucose or T2D and hsa-miR-29b-3p levels (p > 0.05).

## Discussion

The association of PTB and adulthood blood miRNA levels was for the first time studied here in the population based YFS data. Lower levels of hsa-miR-29b-3p were identified in subjects born preterm with either AGA or SGA. Hsa-miR-29b-3p levels were shown to correlate negatively with the mRNA levels of BCL11A and positively with CS mRNA levels and the predicted targets of this specific miRNA were enriched in 13 biological gene sets. The expression of *Alzheimer’s* and *Parkinson’s disease* pathways were negatively associated and the expression of the *Insulin signaling* pathway and *Regulation of actin cytoskeleton* associated positively with hsa-miR-29b-3p levels. We also observed a direct association between the levels of this miRNA and visual processing and sustained attention as well as inverse association with extra-large, large and medium VLDL subclasses and their lipids and triglyceride levels.

Adult (aged 34–49 years) blood levels of hsa-miR-29b-3p, -409-3p and -21-5p were shown here to be associated with individuals birth status. Of these, only the association between hsa-miR-29b-3p levels and birth status remained significant after multiple testing correction. The levels of hsa-miR-29b-3p were shown to be lower in subjects born preterm when comparing to those born full-term and even more so in those born preterm and SGA. This finding is supported by the data from Rager et al.^53^ showing that the two individuals born before the week of 37 had lower miR-29b-3p levels than the median of those who were born at full term. In addition, Pavlek et al. have reported lower levels of miR-29b umbilical cord blood in infants born with spontaneous PTB in comparison to those whose PTB was medically induced. Intriguingly in their data, the 4 individuals born preterm and SGA had higher hsa-miR-29b-3p levels in comparison to those with PTB and AGA^38^. The levels of hsa-miR-29b-3p have been also associated with complications in PTB infants such as bronchopulmonary dysplasia^39^ and adverse events during pregnancy, like preeclampsia^31,32^. All-in-all, the literature describing the associations between circulatory miRNAs and complicated pregnancies or PTB are scare and recent studies have either not analyzed miR-29b-3p or don’t report association between this miRNA and PTB^40,41^.

MicroRNA expression has often been thought to be short-termed and responsive to stimulus. The levels and effect of miRNA in the cytoplasm hindering transcription are seen to be transient. In this light, the association between preterm birth and adulthood miRNA levels could be doubted. Our association does not imply causality and transient hsa-miR-29b-3p levels can be affected by many confounding factors. Hsa-miR-29b-3p is nonetheless a credible candidate for more sustained regulation. Instead of the cytoplasm, miR-29b has been shown to be transported to the nucleus where it can act as a transcriptional or splicing factor^54^. The whole microRNA-29b family has been named epi-miRNAs, as they are capable of controlling epigenetic landscape by targeting key epigenetic effectors, such as DNA methyltransferases and histone deasetylases^55,56^ having longer lasting effects on gene expression. It is also plausible that hsa-miR-29b-3p levels themselves are regulated by pre- and perinatal conditions and/or their consequences as this miRNA has been suggested to have an extensive regulatory role in embryonic development^57,58^. Furthermore, Pavlek et al. reported an association between miR-29b-3p levels and inflammation on preterm infants, suggesting thus a possibility of a link between this miRNA, prenatal life and adulthood health^38^.

The predicted targets of hsa-miR-29b-3p that correlated with its expression were enriched in *Insulin signaling* pathway and the expression of this gene set as a whole was positively associated with hsa-miR-29b-3p levels. Association between miR-29b and T2D is supported by literature^59^. Levels of hsa-miR-29b have been shown to be down-regulated in plasma of individuals who will later develop T2D^60^ and in blood fractions of individuals with either impaired glucose tolerance^61^ or impaired fasting glucose^62^. Additionally, this miRNA has been associated with type 1 and gestational diabetes^28^, and it has been linked to several complications of T2D in small arteries, such as diabetic retinopathy and nephropathy^63–65^. Like us, others have not seen a correlation between fasting glucose^60^ or other indications of sugar metabolism^62^ and hsa-miR-29b-3p levels. Instead, this miRNA has been shown to contribute to insulin resistance and repression of insulin-stimulated glucose uptake in animal-models^66–68^. Interestingly, Dooley et al. have also described the effects of miR-29 family members on both increased insulin signaling in the pancreas and truncating the duration of insulin signaling in the liver^69^, while Jacovetti et al. emphasized the effect of miR-29b-3p on maturation of pancreatic islet cells in newborn rats and the effect of this on later manifestation of metabolic diseases^70^. Both of the predicted target genes of hsa-miR-29b-3p, which levels correlated statistically significantly with the miRNA levels in our data, have also been associated with T2D. BCL11A, which had a negative correlation with hsa-miR-29b-3p levels in our study, has been associated with T2D in genome-wide association studies^66,67^ and in consequence functional studies^73,74^. Alike, CS, which had a positive correlation with hsa-miR-29b-3p levels in our data, has been shown to be down-regulated in the muscle of T2D patients, possibly as a consequence of impaired response to insulin^75,76^.

Our results show a negative association between hsa-miR-29b-3p and VLDL and triglyceride levels. Insulin resistance at the level of the fat cells leads to increased intracellular hydrolysis of triglycerides and release of fatty acids into the circulation. The subsequent excessive flow of fatty acids to the liver results in increased fatty deposition within the hepatocytes and promotes enhanced large VLDL production^77^. This increased secretion of triglyceride-enriched large VLDL particles and elevated plasma triglycerides is commonly seen in patients with insulin resistance and T2D^78^. Dyslipidemia including hypertriglyceridemia is an early event accompanying insulin resistance and precedes beta cell failure^79^. In addition, elevated triglyceride levels have been shown to predict future incidence of T2D independently of traditional risk factors^80^. The miR-29 family has been previously shown to control hepatic lipogenic programs^81^ and dampen de novo synthesis of triglycerides through targeting of Sirtuin-1 in the mouse liver^82^. In cell culture experiments, miR-29b has been shown to target lipoprotein lipase, and thus, to possibly regulate triglyceride production^83^. However, to our knowledge, this is the first study reporting an association between miR-29b and triglyceride levels in humans.

Targets of hsa-miR-29b-3p, which correlated with the miRNA levels in blood, were also enriched in gene sets related to memory (*Long-term potentiation* pathway) and neurodegenerative diseases (*Alzheimer's disease* and *Parkinson’s disease* pathways). Of these, *Alzheimer's disease* and *Parkinson’s disease* pathways expression as a whole were negatively associated with hsa-miR-29b-3p levels. Supporting our findings, down-regulation of hsa-miR-29b-3p has been previously shown in the peripheral blood mononuclear cells^84^, plasma^85^ and brain of subjects^86^ with AD whilst up-regulation of this miRNA has been detected in the cerebrospinal fluid of AD patients^87^. Positive correlations have also been reported between serum mir-29b levels and human cortical thickness and cortical glucose metabolism^88^, which both decrease in AD. Interestingly, pre-mir-29b has even been studied as a potential therapeutic agent targeted against AD^89^.

The expression of *Regulation of acting cytoskeleton* pathway was directly associated with the levels of hsa-miR-29b-3p. Dysregulation of gene sets governing actin cytoskeleton stability leading to synapse loss has been thought to be an early insult in AD^90^. This can be a response to insulin resistance in the brain, possibly being one of the many shared molecular, biochemical and mechanistic abnormalities in T2D and AD^91^. However, even if these two pathologies are known to act in concert, and simultaneously and diabetes is suggested to exacerbate AD pathology, the complex interactions are still not fully understood^92^.

We found a direct association between hsa-miR-29b-3p levels and visual processing and sustained attention (RVP-test), with the strongest associations being observed between total correct rejection and signal detection measure of sensitivity to the target (RVP A´). A significant impairment in this cognitive domain or its individual variables have been previously reported in subjects with AD^93^ or mild cognitive impairment^94^. Though the RVP-test primarily measures processing speed and sustained attention, its successful execution requires also selective attention and working memory i.e. two cognitive subdomains in which impairment has been well documented along with the progression of AD^95^. Importantly, our results are the first to connect hsa-miR-29b-3p to cognitive function in young and healthy adult population without clinically detectable signs of neurodegenerative diseases.

The strength of this study is the birth records and versatile phenotyping of the YFS. As the miRNA profiling has been performed as a cross-sectional study, we cannot infer causality or assess the dynamics of hsa-miR-29b-3p levels during subjects’ lifespans. Previous reports and our analysis show associations between miR-29b-3p levels and PTB^38^. However, as miR-29b levels have been shown to decrease with aging^96^, the differences in the pace of this decrease could also affect the levels in adulthood. Profiling miRNAs from blood poses a challenge for identifying the origin of the miRNAs as blood contains miRNAs from circulatory cells but also circulatory miRNAs originating from various tissues^44^. Whole blood was selected to enable gene-expression analysis from the same sample. Oral glucose tolerance tests had not been performed for the study population, hence we are unable to reflect upon the association between hsa-miR-29b-3p and impaired glucose tolerance. In addition, our analysis does not take account for all occupation-related or other lifestyle factors which affect the later risk of metabolic diseases and dementia or the effects of early life nurture on cognition. Larger replication cohorts are needed to verify the findings. Due to the age of the study populations, there was a low frequency of subjects with T2D and none with cognitive deficits and/or neurodegenerative diseases. Thus, associations between miRNA levels and clinical outcomes were not achievable. However, the observed associations with VLDL levels and cognitive function indicate a possible link to later morbidities.

## Conclusions

Within this study we showed for the first time in humans that adulthood blood levels of hsa-miR-29b-3p were lower in subjects born preterm compared to subjects born full term, and that hsa-miR-29b-3p may be linked in the association between PTB and poor adulthood cognitive function and dyslipidemia. The association of this dysregulated miRNA with cognitive function and metabolic dysfunction could be mediated through its role in regulating gene sets related with neurodegenerative diseases and insulin signaling. We also pointed out that hsa-miR-29b-3p levels were associated with serum VLDL lipid and triglyceride levels, possibly reflecting impaired glucose tolerance which in theory could be related to prediabetes. Similarly, the association between this miRNA and visual processing and sustained attention is linked to the previous findings connecting miR-29b in the pathology of AD. In summary, our results reveal hsa-miR-29b-3p being associated with preterm birth and adulthood metabolic and cognitive health and open new perspectives for pre-clinical investigations.

## Supplementary Information

Supplementary Information 1.

Supplementary Information 2.

## Data Availability

The datasets generated and/or analyzed during the current study are not publicly available. Due to legal restrictions, the Ethics Committee of the Hospital District of Southwest Finland has in 2016 stated that individual level data cannot be stored in public repositories or otherwise made publicly available. However, data are available upon request from YFS Project Application. Data requests can be submitted by email and are subject to approval by the YFS Board.

## Abbreviations

AD: Alzheimer's disease
AGA: Appropriate birth weight for gestational age
BCL1A: BAF chromatin remodeling complex subunit BCL11A
CANTAB: Cambridge Neuropsychological Test Automated Battery
CS: Citrate synthase
FC: Fold change
FDR: False discovery rate
FTB: Full term birth
GSEA: Gene set enrichment analysis
HbA1c: Glycated hemoglobin
HDL: High density lipoprotein
hsa: Homo Sapiens
LDL: Low density lipoprotein
miR: MicroRNA
miRNA: MicroRNA
mRNA: Messenger RNA
NMR: Nuclear magnetic resonance
PAL: Paired associates learning
PTB: Preterm birth
RTI: Reaction time
RVP: Rapid visual information processing
SD: Standard deviation
SGA: Small birth weight for gestational age
SWM: Spatial working memory
T2D: Type 2 diabetes
WHO: World Health Organization
VLDL: Very low-density lipoprotein
YFS: The Young Finns Study
