# Supplementary material for: Adulthood blood levels of hsa-miR-29b-3p associate with preterm birth and adult metabolic and cognitive health
Source: Sci Rep. 2021 Apr 28;11:9203. doi: 10.1038/s41598-021-88465-4 (PMC8080838; doi:10.1038/s41598-021-88465-4)
Supplement: Supplementary file 1 — Supplementary Information 1. [file 41598_2021_88465_MOESM1_ESM.docx]

**Supplementary material**

**Adulthood blood levels of hsa-miR-29b-3p associate with preterm birth and adult metabolic and cognitive health**

Saara Marttila, Suvi Rovio, Pashupati P. Mishra, Ilkka Seppälä, Leo-Pekka Lyytikäinen, Markus Juonala, Melanie Waldenberger, Niku Oksala, Mika Ala-Korpela, Emily Harville, Nina Hutri-Kähönen, Mika Kähönen, Olli Raitakasri, Terho Lehtimäki, Emma Raitoharju

Contents

[**Supplementary figures** 2](#_Toc62028860)

[Supplementary figure 1. 2](#_Toc62028861)

[Supplementary Figure 2 3](#_Toc62028862)

[Supplementary Figure 3 4](#_Toc62028863)

[**Supplementary tables** 5](#_Toc62028864)

[Supplementary table 1. 5](#_Toc62028865)

[Supplementary table 2. 6](#_Toc62028866)

[Supplementary table 4. 7](#_Toc62028867)

[**Supplementary materials and methods** 8](#_Toc62028868)

[The Young Finns Study 8](#_Toc62028869)

[Fetal growth and preterm birth 8](#_Toc62028870)

[RNA isolation and quality control 8](#_Toc62028871)

[MicroRNA expression profiling 8](#_Toc62028872)

[Genome-wide expression analysis 9](#_Toc62028873)

[Clinical and biochemical measurements 9](#_Toc62028874)

[NMR metabolomics 9](#_Toc62028875)

[Cognitive function 9](#_Toc62028876)

[Statistical analysis 10](#_Toc62028877)

[Supplementary references 12](#_Toc62028878)

# **Supplementary figures**

## Supplementary figure 1.

Flow and results of the target and pathway analysis performed.


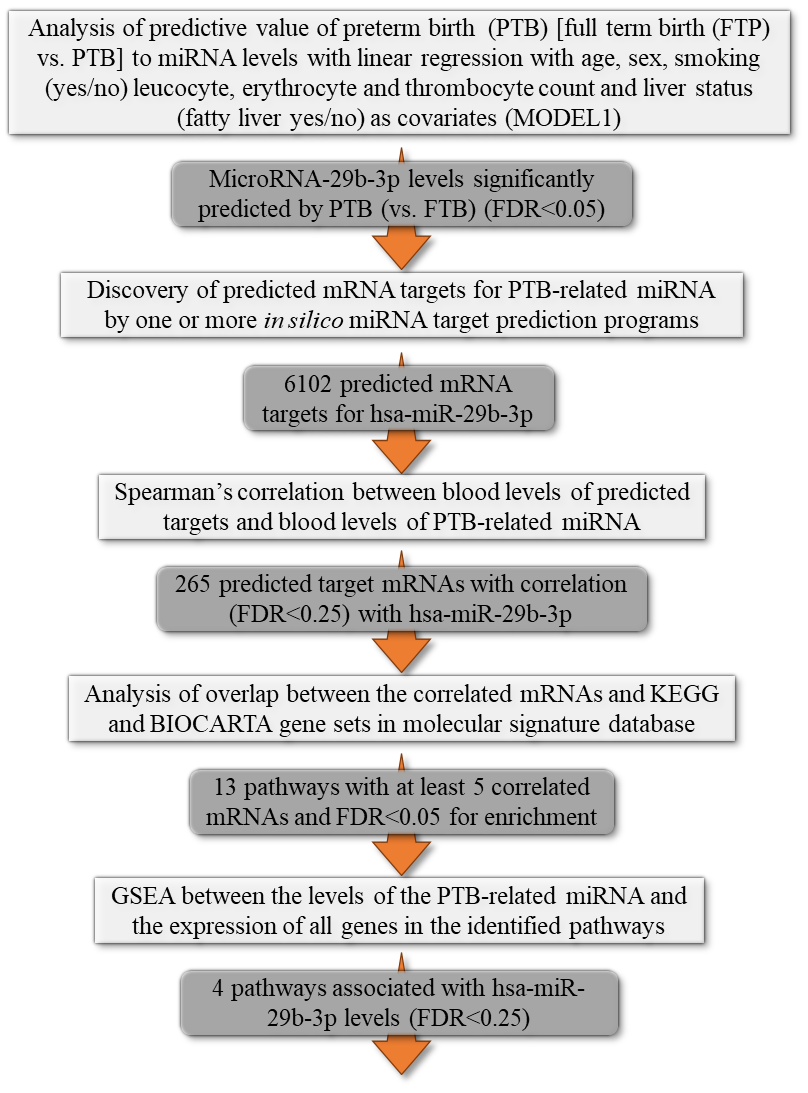


Abbreviations: PTB = preterm birth, FTP= full term birth, FDR=false discovery rate

Supplementary Figure 2**.** Blood levels of hsa-miR-29b-3p of women (A) and men (B) separately born preterm and either appropriate birth weight for gestational age (AGA) or small birth weight for gestational age (SGA) described by the Log2(fold changes) in comparison to subjects born full-term.

B.

A.

Statistics: = Mann-Whitneyn U-test between subjects born full-term and preterm groups.

= Kruskal–Wallis test over all groups

Supplementary Figure 3**.** Blood levels of hsa-miR-29b-3p of newborn infants in the Biomarkers of Exposure to ARsenic (BEAR) pregnancy cohort(1) described by the Log2(fold changes) comparing newborns born preterm to those born full-term (Fold change between groups is -1.81).

Statistics: = Mann-Whitneyn U-test between subjects born full-term and preterm.

# **Supplementary tables**

## Supplementary table 1.

Demographic of the study population. Continuous variables are presented as medians and standard deviations in brackets.

|  |  | **All** | **Full-term** | **Preterm, AGA** | **Preterm, SGA** |
| --- | --- | --- | --- | --- | --- |
| Number of subjects |  | 765 | 681 | 67 | 17 |
| Age, years |  | 43 (4.8) | 43 (4.8) | 43.0 (4.7) | 43.0 (3.9) |
| Males, (%) |  | 343 (44.8) | 308 (45.2) | 30 (44.8) | 5 (29.4) |
| Total cholesterol, mmol/l |  | 5.0 (0.9) | 5.0 (0.9) | 5.1 (0.8) | 5.0 (0.9) |
| HDL cholesterol, mmol/l |  | 1.3 (0.3) | 1.3 (0.3) | 1.3 (0.3) | 1.3 (0.4) |
| LDL cholesterol, mmol/l |  | 3.1 (0.8) | 3.2 (0.8) | 3.1 (0.7) | 2.7 (0.7 |
| Triglycerides, mmol/l |  | 1.1 (0.7) | 1.1 (0.7) | 1.0 (0.8) | 1.2 (1.0) |
| Type 2 diabetes, (%) |  | 19 (2.7) | 16 (2.5) | 3 (4.5) | 0 (0) |
| Impaired fasting glucose, (%)* |  | 219 (30.8) | 186 (29.5) | 24 (35.8) | 9 (52.9) |
| Blood glucose, mmol/l |  | 5.3 (0.8) | 5.3 (0.9) | 5.3 (0.5) | 5.4 (0.3) |
| HbA1c, %, |  | 5.4 (0.4) | 5.4 (0.4) | 5.5 (0.3) | 5.5 (0.3) |
| HbA1c, mmol/mol |  | 36.0 (4.2) | 36.0 (4.3) | 37.0 (3.7) | 36.0 (3.6) |
| Insulin, mU/L* |  | 7.3 (16.2) | 7.1 (17.0) | 7.9 (7.2) | 9.1 (6.6) |
| Hypertension, (%) |  | 58 (8.0) | 53 (8.3) | 5 (7.5) | 0 (0) |
| Body mass index, kg/m2 |  | 25.6 (4.8) | 25.6 (4.9) | 25.5 (4.4) | 28.3 (3.8) |
| Birth weight, kg* |  | 3.6 (0.5) | 3.6 (0.5) | 3.2 (0.6) | 2.2 (0.5) |
| Fatty liver, (%) |  | 120 (15.7) | 104 (15.3) | 12 (17.9) | 4 (23.5) |
| Erythrocyte count, 10^12^/L |  | 4.7 (0.4) | 4.7 (0.4) | 4.8 (0.4) | 4.6 (0.4) |
| Leucocyte count, 10^9^/L |  | 5.3 (1.5) | 5.3 (1.5) | 5.4 (1.5) | 5.3 (1.5) |
| Thrombocyte count, 10^9^/L* |  | 254.0 (58.7) | 253.0 (59.6) | 274.0 (51.2) | 253.0 (59.6) |
|  |  |  |  |  |  |

^*^p<0.05 over the birth status groups

Supplementary table 2. MicroRNAs nominally significantly predicted by preterm birth (full term birth vs. preterm birth) in linear regression with age, sex, smoking (yes/no), serum insulin and glucose levels, leucocyte, erythrocyte and thrombocyte count and liver status (fatty liver yes/no) as covariates. The β-value describes the standard deviation increment in miRNA levels when comparing subjects with PTB to those born full term.

| **MicroRNA** | **n** | **p-value** | **FDR** | **β** | **95% CI** |
| --- | --- | --- | --- | --- | --- |
| hsa-miR-29b-3p | 551 | 1.90*10^-4^ | 0.046 | -0.521 | (-0.792 - -0.249) |
| hsa-miR-409-3p | 723 | 0.004 | 0.526 | -0.345 | (-0.582- -0.108) |
| hsa-miR-21-5p | 730 | 0.009 | 0.729 | -0.308 | (-0.539- -0.078) |

**Abbreviations:** FDR, False discovery rate.

Supplementary table 4. Associations of hsa-miR-29b-3p with metabolite levels as well as physiological features previously associated with metabolic dysfunction. If not otherwise stated, the metabolite has been measured with NMR metabolomics. In the regression model **(MODEL2)** the Betas (β) indicate the standard deviation (SD) change of the metabolite levels per increase of one SD of miRNA levels.

| **Metabolite** | **p-value** | **FDR** | **β** | **95%CI** |
| --- | --- | --- | --- | --- |
| M VLDL lipids | 0.001 | 0.020 | -0.128 | (-0.202 - -0.054) |
| M VLDL phospholipids | 0.001 | 0.020 | -0.126 | (-0.200 - -0.052) |
| VLDL triglycerides | 0.001 | 0.020 | -0.124 | (-0.197 - -0.051) |
| M VLDL particles | 0.001 | 0.020 | -0.125 | (-0.198 - -0.051) |
| M VLDL free cholesterol | 0.001 | 0.020 | -0.126 | (-0.200 - -0.052) |
| M VLDL cholesterol | 0.001 | 0.020 | -0.126 | (-0.202 - -0.051) |
| XL VLDL triglycerides | 0.001 | 0.020 | -0.114 | (-0.182 - -0.045) |
| M VLDL triglycerides | 0.001 | 0.020 | -0.122 | (-0.195 - -0.048) |
| L VLDL cholesterol esters | 0.001 | 0.020 | -0.115 | (-0.184 - -0.045) |
| Triglycerised to phospholipids | 0.002 | 0.020 | -0.117 | (-0.189 - -0.045) |
| S VLDL triglycerides | 0.002 | 0.020 | -0.118 | (-0.191 - -0.045) |
| XL VLDL particles | 0.002 | 0.020 | -0.109 | (-0.176 - -0.041) |
| M VLDL cholesterol esters | 0.002 | 0.020 | -0.123 | (-0.199 - -0.047) |
| XL VLDL lipids | 0.002 | 0.020 | -0.110 | (-0.179 - -0.042) |
| L VLDL cholesterol | 0.002 | 0.021 | -0.112 | (-0.183 - -0.042) |
| Serum triglycerides | 0.002 | 0.021 | -0.118 | (-0.193 - -0.043) |
| VLDL triglycerides ^A^ | 0.002 | 0.021 | -0.116 | (-0.190 - -0.042) |
| L VLDL particles | 0.002 | 0.022 | -0.114 | (-0.187 - -0.040) |
| S VLDL particles | 0.003 | 0.022 | -0.113 | (-0.187 - -0.040) |
| L VLDL lipids | 0.003 | 0.023 | -0.110 | (-0.183 - -0.038) |
| L VLDL triglycerides | 0.003 | 0.023 | -0.111 | (-0.184 - -0.038) |
| L VLDL free cholesterol | 0.003 | 0.023 | -0.107 | (-0.178 - -0.036) |
| Triglycerides ^B^ | 0.003 | 0.023 | -0.111 | (-0.185 - -0.037) |
| S VLDL lipids | 0.004 | 0.024 | -0.110 | (-0.184 - -0.036) |
| VLDL diameter | 0.005 | 0.032 | -0.106 | (-0.180 - -0.032) |
| S VLDL phospholipids | 0.005 | 0.032 | -0.107 | (-0.181 - -0.032) |
| L VLDL phospholipids | 0.005 | 0.032 | -0.104 | (-0.178 - -0.031) |
| HDL3 cholesterol | 0.005 | 0.032 | -0.118 | (-0.200 - -0.035) |
| S VLDL free cholesterol | 0.006 | 0.033 | -0.105 | (-0.180 - -0.031) |
| XL VLDL phospholipids | 0.007 | 0.035 | -0.097 | (-0.166 - -0.027) |
| CH2 groups of mobile lipids | 0.008 | 0.040 | -0.106 | (-0.184 - -0.028) |
| XXL VLDL lipids | 0.010 | 0.050 | -0.091 | (-0.161 - -0.022) |
| XS VLDL triglycerides | 0.011 | 0.053 | -0.101 | (-0.179 - -0.024) |
| XXL VLDL triglycerides | 0.014 | 0.068 | -0.087 | (-0.157 - -0.018) |
| S VLDL cholesterol | 0.015 | 0.068 | -0.094 | (-0.170 - -0.019) |
| XXL VLDL phospholipids | 0.015 | 0.068 | -0.086 | (-0.156 - -0.017) |
| Bisallylic groups per total fatty acids | 0.017 | 0.074 | 0.093 | (0.017 - 0.169) |
| Total fatty acid | 0.018 | 0.079 | -0.096 | (-0.175 - -0.016) |
| Monounsaturated fatty acids | 0.019 | 0.079 | -0.094 | (-0.172 - -0.016) |
| Douple bonds per fatty acids | 0.020 | 0.082 | 0.090 | (0.014 - 0.166) |
| Omega-9 and saturated fatty acids | 0.023 | 0.089 | -0.090 | (-0.168 - -0.013) |
| Bisallylic groups per double bond | 0.023 | 0.089 | 0.090 | (0.013 - 0.167) |
| IDL cholesterol ^A^ | 0.024 | 0.091 | -0.086 | (-0.161 - -0.012) |
| S HDL trilycerides | 0.025 | 0.092 | -0.086 | (-0.161 - -0.011) |
| Apolipoprotein B | 0.028 | 0.101 | -0.086 | (-0.162 - -0.010) |
| CH3 groups of mobile lipids | 0.030 | 0.105 | -0.090 | (-0.171 - -0.009) |
| XS VLDL particles | 0.030 | 0.105 | -0.090 | (-0.171 - -0.009) |
| XXL VLDL particles | 0.031 | 0.105 | -0.082 | (-0.155 - -0.008) |
| Average fatty acid chain length | 0.032 | 0.105 | 0.091 | (0.008 - 0.174) |
| Omega-6 fatty acids | 0.038 | 0.125 | -0.090 | (-0.174 - -0.005) |
| Apolipoprotein B ^B^ | 0.045 | 0.145 | -0.079 | (-0.157 - -0.002) |
| XS VLDL lipids | 0.047 | 0.147 | -0.083 | (-0.165 - -0.001) |

^A^ Calculated, ^B^ Enzymatically measured

Statistical model MODEL2=linear regression with hsa-miR-29b-3p predicting metabolite levels one by one with age, sex, smoking (yes/no), birth status (full term birth/AGA/SGA), leucocyte, erythrocyte and thrombocyte count, polygenic risk score for metabolic syndrome and liver status (fatty liver yes/no) as covariates.

# **Supplementary materials and methods**

## The Young Finns Study

YFS is a multicenter follow-up study on cardiovascular risk from childhood to adulthood in Finland. The YFS was launched in 1980, when 3,596 children and adolescents (3–18 years old) participated in the baseline study39. Thereafter, the subjects have been followed up with several examinations including comprehensive risk factor assessments. The 30-year follow-up was performed in 2011, with 2,063 adults, aged 34–49 years, participating in the study. The examinations included physical measurements, blood tests, and questionnaires. The present study has been approved by the 1st ethical committee of the Hospital District of Southwest Finland on September 21st, 2010 and by local ethical committees. All study subjects gave an informed consent, and the study was conducted according to the principles of the Declaration of Helsinki. All measurements utilized in this study, excluding birthweight and prematurity at birth, are from the 2011 follow-up and samples collected during it. The study population of this study comprises 871 subjects from 2011 follow-up with successful miRNA profiling. When the population in the miRNA analysis was compared to the whole 2011 follow-up study population, the only differences were observed in the prevalence of T2D, which in the whole population is 6.0% and in the microRNA profiling cohort only 3.0%. Birth weight and preterm status were available for 761 subjects in the microRNA profiling sub-population. The demographics are presented in Supplementary table 1.

## Fetal growth and preterm birth

Preterm birth status was defined as birth before 37 weeks’ gestation, and the number of weeks preterm was ascertained in those who reported preterm birth. Birthweight was reported during the follow-up studies in 1983 and 1986. Subjects born preterm were categorized as either AGA or with SGA using a cut point of − 1 SD z score (corresponding to the 15th percentile) based on Finnish sex and gestational age-stratified birthweight percentiles (2). AGA describes a newborn infant whose size is within the normal range for his or her gestational age, while newborns with SGA were smaller than expected for their gestational weeks. Study population did not include subjects born preterm and large for their gestational age.

## RNA isolation and quality control

Whole blood (2.5ml) was collected into PaXgene Blood RNA Tubes (PreAnalytix). The tubes were inverted 8–10 times then stored at room temperature for at least 2 hours. The tubes were frozen (-80°C) and thawed overnight before RNA isolation (both miRNA and total RNA) with a PAXgene Blood microRNA Kit (Qiagen) including the DNase Set using the QiaCube. The concentrations and purity of the RNA samples were evaluated spectrophotometrically (BioPhotomer, Eppendorf). The RNA isolation process was validated by analyzing the integrity of several RNAs with the RNA 6000 Nano Chip Kit (Agilent). The presence of the small RNA fraction was confirmed by the Agilent Small RNA Kit (Agilent).

## MicroRNA expression profiling

MicroRNA expression profiling was performed with the TaqMan® OpenArray® MicroRNA Panel (Applied Biosystems) containing 758 microRNAs. Briefly, 100 ng of RNA was used to run both A and B pools of Megaplex (Applied biosystems) preamplification for cDNA synthesis. In the OpenArray Sample Loading Plate, 22.5 µl of each preamplified pool was mixed 1:1 with TaqMan OpenArray Real-Time PCR Master Mix. MicroRNA panels were loaded using the AccuFill System and run with the QuantStudio 12K Flex (Applied Biosystems).

Primary data analysis was performed with Expression Suite Software version 1.0.1. U6 snRNA, RNU44, and RNU48 were used as housekeeping small RNAs. Assays with Amplification score >1 and Cq Confidence >0.7 were accepted. Ninety-five samples were excluded due to a low number of miRNAs expressed (≤200 miRNAs per sample), and in further analysis, 243 miRNAs that were expressed in at least 2/3 of the samples were included. The RNA quality and functionality of the TaqMan OpenArray microRNA expression panels have been validated previously(3). After quality control and removal of outlier miRNA, profiling was successful on 871 samples. To correct for batch effects, the principal component analysis was performed for the miRNA expression data. The data was adjusted for 10 of the first 20 principal components from the principal component analysis.

## Genome-wide expression analysis

The expression levels were analyzed with an Illumina HumanHT-12 version 4 Expression BeadChip (Illumina Inc.). In brief, 200 ng of RNA was reverse-transcribed into cDNA and biotin-UTP-labeled using the Illumina TotalPrep RNA Amplification Kit (Ambion); 1,500 ng of cDNA was then hybridized to the Illumina HumanHT-12 v4 Expression BeadChip. The BeadChips were scanned with the Illumina iScan system. Raw Illumina probe data was exported from Genomestudio and analyzed in R (http://www.r-project.org/) using the Bioconductor (http://www.bioconductor.org/) packages. The expression data was processed using nonparametric background correction, followed by quantile normalization with control and expression probes, using the neqc function in the limma package and log2 transformation. The expression analysis was successful in 743 of the 871 samples with a miRNA expression profile.

## Clinical and biochemical measurements

Weight and height were measured and BMI calculated as weight(kg)/(height(m))². Waist circumference was measured to the nearest 0.1 cm. Blood pressure was measured with a random zero sphygmomanometer*.*

Venous blood samples were drawn from the right antecubital vein after an overnight fast. For blood count analysis, whole blood was anticoagulated with EDTA. For the biochemical measurements, serum was separated, aliquoted, and stored at -70°C until analysis. The serum triglyceride concentration was assayed using the enzymatic glycerol kinase–glycerol phosphate oxidase method (Beckman Coulter Biomedical). Serum total cholesterol levels were measured by the enzymatic cholesterol esterase–cholesterol oxidase method (Beckman Coulter Biomedical). The same reagent was used for estimating high-density lipoprotein (HDL) cholesterol levels after the precipitation of apolipoprotein B-containing lipoproteins with dextran sulfate- Mg^2+^. Serum glucose concentrations were determined by the enzymatic hexokinase method (Beckman Coulter Biomedical). All the above-mentioned assays were performed on an AU400 instrument (AU400, Olympus).

For glycated hemoglobin (HbA1c) fraction measurement, the concentration of total hemoglobin was determined colorimetrically, after which the concentration of HbA1c was measured immunoturbidimetrically. These two concentrations were used to calculate the HbA1c percentage (HbA1c%). Insulin levels were measured by a microparticle enzyme immunoassay kit. Subjects were categorized into the normoglycemic, impaired fasting glucose and T2D groups. The classification was based on fasting serum glucose and HbA1c according to the criteria of the WHO(4) and self-reported diagnosis of T2D by a physician. Subjects with type 1 diabetes were discarded from the analysis.

## NMR metabolomics

A high-throughput serum NMR metabolomics platform was used for absolute quantification of serum lipids and metabolites, including lipoprotein subclass distributions, fatty acids, and various small molecules such as amino acids and glycolysis precursors(5,6). The analyzed 14 lipoprotein subclasses were defined based on particle size. These detailed lipoprotein subclass measurements, together with standard total lipids and apolipoproteins, provide a good basis for studying the lipid- and lipoprotein-related metabolic pathways. The NMR-based metabolic profiling has previously been used in various epidemiological and genetic studies(5), and details of the experimentation have been described(5–7). Data were available from all the subjects with successful miRNA profiling.

## Cognitive function

A test battery developed by the Cambridge Cognition (CANTAB®) was used to assess cognitive function among the subjects in the follow-up study in 2011. The CANTAB® test is a computerized, predominantly non-linguistic and culturally neutral test focusing on a wide range of cognitive domains. The test was performed using a validated touch-screen computer system and was successful on all the 871 participants with microRNA expression data. In YFS, the test battery was compiled and performed as described earlier47. During cognitive testing the participants conducted a Motor screening test, Paired associates learning test, Spatial working memory test, Reaction time test and Rapid visual information processing test. Each test produced several variables. Principal component analysis was conducted for each test to identify components accounting for the majority of the variation within the dataset. The first principal component was selected to represent the performance in each separate test. After distribution analyses, the Motor screening test component was excluded from further analyses due to ceiling effect. Other components were normalized using rank order normalization procedure resulting in four variables (mean 0, standard deviation (SD) 1)(8).

## Statistical analysis

If not otherwise stated, all analyses were performed with R-language and all continuous variables were inverse normal transformed in regression models. The predictive value of PTB status (full term birth vs. preterm birth) to adulthood miRNA levels was analyzed with linear regression with age, sex, smoking (yes/no), ), serum insulin and glucose levels, leucocyte, erythrocyte and thrombocyte count and liver status (fatty liver yes/no) as covariates **(MODEL 1).** To account for multiple testing, only miRNAs with FDR<0.05 were further investigated. Median fold changes (FC) between different birth status groups (FTB, PTB with AGA or PTB with SGA) were calculated their and pairwise statistical significance was evaluated with Mann-Whitney U test and over all groups using Kruskal-Wallis test for trend.

The flow and results of transcriptomic and gene set analysis are presented in Supplementary figure 1. The miRNA-target mRNA analysis and target selection are independent of the regression models and target selection/correlations are not adjusted with cofounding factors. The predicted mRNA targets of miRNAs of interest were included in the correlation analysis if they were recognized by one or more *in silico* miRNA target prediction programs in miRGator. Correlations were calculated with Spearman’s correlation and individual correlations with FDR<0.05 (both positive and negative correlations) were considered significant. Predicted target mRNAs with correlation at the level of FDR<0.25 with the levels miRNA of interest were selected in further analysis. The overlaps between the selected target mRNAs and gene set in KEGG and BIOCARTA were analyzed in molecular signature database (www.gsea-msigdb.org/gsea/msigdb/index.jsp). Gene sets containing at least 5 of the selected target mRNAs and with FDR<0.05 for the enrichment were included in gene set enrichment analysis (GSEA). In GSEA (adjusted with age and sex), the association between the expression of all genes in the identified gene sets and blood levels of the miRNA of interest were evaluated and FDR<0.25 was considered significant.

Association between the miRNAs of interest and the metabolite levels as well as physiological features previously associated with metabolic dysfunction (listed in 42) were analyzed one by one with linear regression analysis using age, sex, smoking, birth status, leucocyte, erythrocyte and thrombocyte count, liver status and polygenic risk score for metabolic syndrome (9) as covariates (**MODEL 2**). Associations between the miRNA and diagnosis of hypertension, T2D and impaired fasting glucose were analysed separately with binominal regression models using the covariates according to the **MODEL 2**.

The associations between the miRNA of interest and each studied cognitive domain were analyzed separately with linear regression with age, sex, smoking, birth status, leucocyte, erythrocyte and thrombocyte count and liver status as well as parental education years (less than 9 y., 9-12 y. or more than 12 y.), participants own education level (participated in academic education yes/no) and polygenic risk score for cognitive function (see below) as covariates (**MODEL 3**). If the miRNA of interest associated significantly (FDR<0.05) with any of the cognitive domains, its association with the individual variables produced by the specific cognitive test for that particular cognitive domain was also analyzed one by one utilizing the adjustments according to the **MODEL 3**. FDR<0.05 was considered significant when utilizing models 1, 2 and 3.

For the polygenic risk for cognitive function, enotyping was performed for 2443 samples using custom build Illumina Human 670k BeadChip at Welcome Trust Sanger Institute. Genotypes were called using Illuminas clustering algorithm(10). Genotype imputation was done using Beagle software(11) and The Sequencing Initiative Suomi (SISu) as reference data. A polygenic risk score for cognitive performance (hereafter polygenic risk score) was calculated using LDpred, a Bayesian method that estimates posterior mean causal effect sizes from genome wide association study (GWAS) summary statistics by assuming a prior for the genetic architecture and linkage disequilibrium (LD) information from a reference panel(12): an infinitesimal fraction of causal variants was assumed and summary statistics from Savage et al. (13) GWAS for intelligence were used. The LD between markers was estimated from the SISu data.

## Supplementary references

1. Rager JE, Bailey KA, Smeester L, Miller SK, Parker JS, Laine JE, et al. Prenatal arsenic exposure and the epigenome: Altered microRNAs associated with innate and adaptive immune signaling in newborn cord blood. Environ Mol Mutagen. 2014;

2. Skilton MR, Viikari JS, Juonala M, Laitinen T, Lehtimaki T, Taittonen L, et al. Fetal growth and preterm birth influence cardiovascular risk factors and arterial health in young adults: the Cardiovascular Risk in Young Finns Study. Arterioscler Thromb Vasc Biol. 2011;31(12):2975–81.

3. Raitoharju E, Seppala I, Oksala N, Lyytikainen LP, Raitakari O, Viikari J, et al. Blood microRNA profile associates with the levels of serum lipids and metabolites associated with glucose metabolism and insulin resistance and pinpoints pathways underlying metabolic syndrome: The cardiovascular risk in Young Finns Study. Mol Cell Endocrinol. 2014;391(1–2):41–9.

4. Organization WH. Global report on diabetes. Geneva. 2016.

5. Soininen P, Kangas AJ, Wurtz P, Tukiainen T, Tynkkynen T, Laatikainen R, et al. High-throughput serum NMR metabonomics for cost-effective holistic studies on systemic metabolism. Analyst. 2009;134(9):1781–5.

6. Soininen P, Kangas AJ, Wurtz P, Suna T, Ala-Korpela M. Quantitative serum nuclear magnetic resonance metabolomics in cardiovascular epidemiology and genetics. Circ Genet. 2015;8(1):192–206.

7. Inouye M, Kettunen J, Soininen P, Silander K, Ripatti S, Kumpula LS, et al. Metabonomic, transcriptomic, and genomic variation of a population cohort. Mol Syst Biol. 2010;6:441.

8. Rovio SP, Pahkala K, Nevalainen J, Juonala M, Salo P, Kahonen M, et al. Cognitive Performance in Young Adulthood and Midlife: Relations With Age, Sex, and Education-The Cardiovascular Risk in Young Finns Study. Neuropsychology. 2015;

9. Kristiansson K, Perola M, Tikkanen E, Kettunen J, Surakka I, Havulinna AS, et al. Genome-wide screen for metabolic syndrome susceptibility Loci reveals strong lipid gene contribution but no evidence for common genetic basis for clustering of metabolic syndrome traits. Circ Genet. 2012;5(2):242–9.

10. Teo YY, Inouye M, Small KS, Gwilliam R, Deloukas P, Kwiatkowski DP, et al. A genotype calling algorithm for the Illumina BeadArray platform. Bioinformatics. 2007;

11. Browning BL, Browning SR. A unified approach to genotype imputation and haplotype-phase inference for large data sets of trios and unrelated individuals. Am J Hum Genet. 2008;

12. Vilhjálmsson BJ, Yang J, Finucane HK, Gusev A, Lindström S, Ripke S, et al. Modeling Linkage Disequilibrium Increases Accuracy of Polygenic Risk Scores. Am J Hum Genet. 2015;

13. Savage JE, Jansen PR, Stringer S, Watanabe K, Bryois J, De Leeuw CA, et al. Genome-wide association meta-analysis in 269,867 individuals identifies new genetic and functional links to intelligence. Nat Genet. 2018;
